# Improving Dental Experiences by Using Virtual Reality Distraction: A Simulation Study

**DOI:** 10.1371/journal.pone.0091276

**Published:** 2014-03-12

**Authors:** Karin Tanja-Dijkstra, Sabine Pahl, Mathew P. White, Jackie Andrade, Cheng Qian, Malcolm Bruce, Jon May, David R. Moles

**Affiliations:** 1 School of Psychology, Plymouth University, Plymouth, United Kingdom; 2 European Centre for Environment and Human Health, University of Exeter Medical School, Truro, United Kingdom; 3 School of Electronic, Electrical, and Computer Engineering, University of Birmingham, Birmingham, United Kingdom; 4 Plymouth University Peninsula Schools of Medicine and Dentistry, Plymouth, United Kingdom; ICREA-University of Barcelona, Spain

## Abstract

Dental anxiety creates significant problems for both patients and the dental profession. Some distraction interventions are already used by healthcare professionals to help patients cope with unpleasant procedures. The present study is novel because it a) builds on evidence that natural scenery is beneficial for patients, and b) uses a Virtual Reality (VR) representation of nature to distract participants. Extending previous work that has investigated pain and anxiety during treatment, c) we also consider the longer term effects in terms of more positive memories of the treatment, building on a cognitive theory of memory (Elaborated Intrusions). Participants (n = 69) took part in a simulated dental experience and were randomly assigned to one of three VR conditions (active vs. passive vs. control). In addition, participants were distinguished into high and low dentally anxious according to a median split resulting in a 3×2 between-subjects design. VR distraction in a simulated dental context affected memories a week later. The VR distraction had effects not only on concurrent experiences, such as perceived control, but longitudinally upon the vividness of memories after the dental experience had ended. Participants with higher dental anxiety (for whom the dental procedures were presumably more aversive) showed a greater reduction in memory vividness than lower dental-anxiety participants. This study thus suggests that VR distractions can be considered as a relevant intervention for cycles of care in which people’s previous experiences affect their behaviour for future events.

## Introduction

Patient pain and anxiety are undesirable side-effects of many medical procedures and can affect the patient’s willingness to undergo treatment [1], [2]. Medical (e.g. analgesic) interventions to reduce pain during treatments are frequently used but can be expensive and may have their own side-effects. Simple, non-invasive alternatives, such as “distraction therapy” are therefore desirable. The use of virtual reality (VR) as a distraction tool is receiving growing attention in medical contexts.

Distraction is thought to help patients cope with pain and other aversive experiences and is often combined with relaxation or pleasant imagery [3], although the psychological mechanisms underlying its effects are not well understood [4]. VR distraction during aversive experiences can improve coping with pain [5], lower experienced level of itching for chronic puritis patients [6], and reduce the perceived duration of procedures [7]. A recent systematic review of eleven studies looked at the effectiveness of virtual reality distraction on pain reduction [8]. They concluded that more sophisticated VR techniques, capable of completely immersing the individual were associated with greater pain relief. According to Gold and colleagues [9] VR provides a powerful means of modifying affect, because of its immersive nature.

Most previous work has considered the effects of VR distraction on pain and anxiety during treatment. Distraction may also have lasting effects in terms of more positive memories of the treatment, leading to a greater willingness to return for treatment. The aim of the current study was to study both immediate and more long-term effects of VR distraction in a simulated dental context. We chose a simulated rather than real treatment for ethical reasons, as we wanted to include participants high in dental anxiety, for whom a simulated treatment would be stressful already.

Dentistry has received relatively little attention from VR researchers, yet it is one of the most common healthcare encounters. Dental anxiety is very common [10] and anxious patients are less likely to keep their appointments [11], take longer to treat and feel less satisfied with their treatment [12], and make their dentists feel anxious too [13]. Armfield and colleagues [14] described a vicious cycle of dental anxiety. This suggests that people with high dental fear delay dental treatment, which can lead to more extensive dental problems and symptomatic visiting patterns which in turn maintain or exacerbate existing dental fear. Memories and expectations thus play a crucial role in sustaining dental anxiety. Although we focus on dental treatment, experiences and expectancies are very important in determining future uptake of treatment in a range of medical contexts, e.g., unpleasant bowel examinations [15].

VR distraction during dental treatment may improve the treatment experience and, by doing so, help break the cycle of negative experiences leading to negative memories and expectations about future treatment. The Elaborated Intrusion theory [16] argues that unconscious cognitive activity triggered by cues in the world, mind or body can lead to apparently spontaneous intrusive thoughts, and that salience of the intrusion can lead to the thought being elaborated, through the construction of mental imagery. Heightened emotion and arousal during a dental examination will increase the likelihood of recollections of the event being triggered uncontrollably by situational cues [17], as a whiff of antiseptic might trigger thoughts about dental treatment. Attempts at suppressing these intrusive thoughts tend to be counterproductive [18], and once triggered, intrusive thoughts tend to be elaborated [16]. For example, an intrusive thought about going to the dentist might lead to the patient imagining how uncomfortable the next visit is going to be and experiencing some of the negative sensations and emotions they associate with dental treatment. Interfering with the processing of negative stimuli during treatment, through VR distraction, would counteract the effects of heightened emotion and arousal and so reduce the likelihood of intrusive thoughts and negative elaborations following treatment. Additionally, it would be desirable to identify if VR distraction is a suitable technique for patients with all levels of dental anxiety or whether specific patients would be most likely to benefit. We therefore included level of dental anxiety as a moderating variable.

There have been a few studies of VR in a dental context. A case study showed that VR distraction is more effective in offering pain control than watching a video or a standard care situation without distraction [19]. One study investigated the effects of using an A/V eyeglass system displaying an instructional video [20]. Adult patients scheduled for dental prophylaxis were distracted during half of their treatment. Patients reported less anxiety and discomfort when using the equipment. In another study, patients undergoing periodontal scaling and root planning procedures were presented with either a control situation (only wearing the headgear), a video (i.e. the animation movie Cars) and a VR environment (of a botanical garden in Second Life) [21]. Both distracters, relative to the control condition, resulted in less pain and discomfort and lower blood pressure and pulse rate, but the VR environment was better on all indicators compared to the movie. This difference can possibly be explained by looking at the level of interactivity VR distraction offers compared to passively watching a video.

Dahlquist and colleagues [5] tested the role of interactivity more directly, by assessing pain tolerance and pain threshold in children using the cold pressor task. In a within-subjects design, the children played a computer game, Finding Nemo, and watched a video of someone else playing the exact same computer game. Both types of distraction reduced pain threshold, but pain tolerance was almost twice as long during interactive distraction relative to passive distraction. The authors suggested that the interactive distraction involved two additional sensory attentional pathways and that the game required problem-solving, providing an active cognitive processing component.

The current study used a VR environment of a coastal nature area to distract participants during simulated dental treatment. This environment was chosen as previous research demonstrated the beneficial effects of nature [22], [23], in particular coastal environments [24], [25]. We investigated whether offering such distraction improved the dental experience both immediately and a week later. We also investigated active versus passive use of the same VR environment and the role of pre-existing dental anxiety. One of the concerns with VR distraction is that it might affect patient-clinician communication. Therefore we tested whether the VR interfered with this by recording compliance with the dentist’s requests.

In terms of overall experience, first we hypothesized that providing VR distraction during simulated dental treatment would result in lower time perception compared to no VR distraction, based on previous research suggesting that the use of VR can affect time perception [7]. Second, in accordance with EI theory, we proposed that offering VR distraction results in less vivid memories and less intrusive thoughts a week later.

The second set of hypotheses focussed on the comparison of active and passive VR. We predicted that the active VR group would experience a higher level of control (manipulation check) and a higher level of presence. Third, we predicted that the effects for the overall experience both immediately and after a week would be stronger for the active VR group compared to the passive VR group.

The third set of hypotheses proposed that pre-existing dental anxiety would moderate these effects. We hypothesized that the effects for the dental experience, the VR experience, and the follow-up effects, would be more pronounced for participants higher in dental anxiety.

## Method

### Ethics statement

The study was approved by the Faculty of Science and Technology ethics review board, Plymouth University. Participants signed a consent form prior to participating, which was approved by the ethics review board.

### Participants

**Figure 1:**
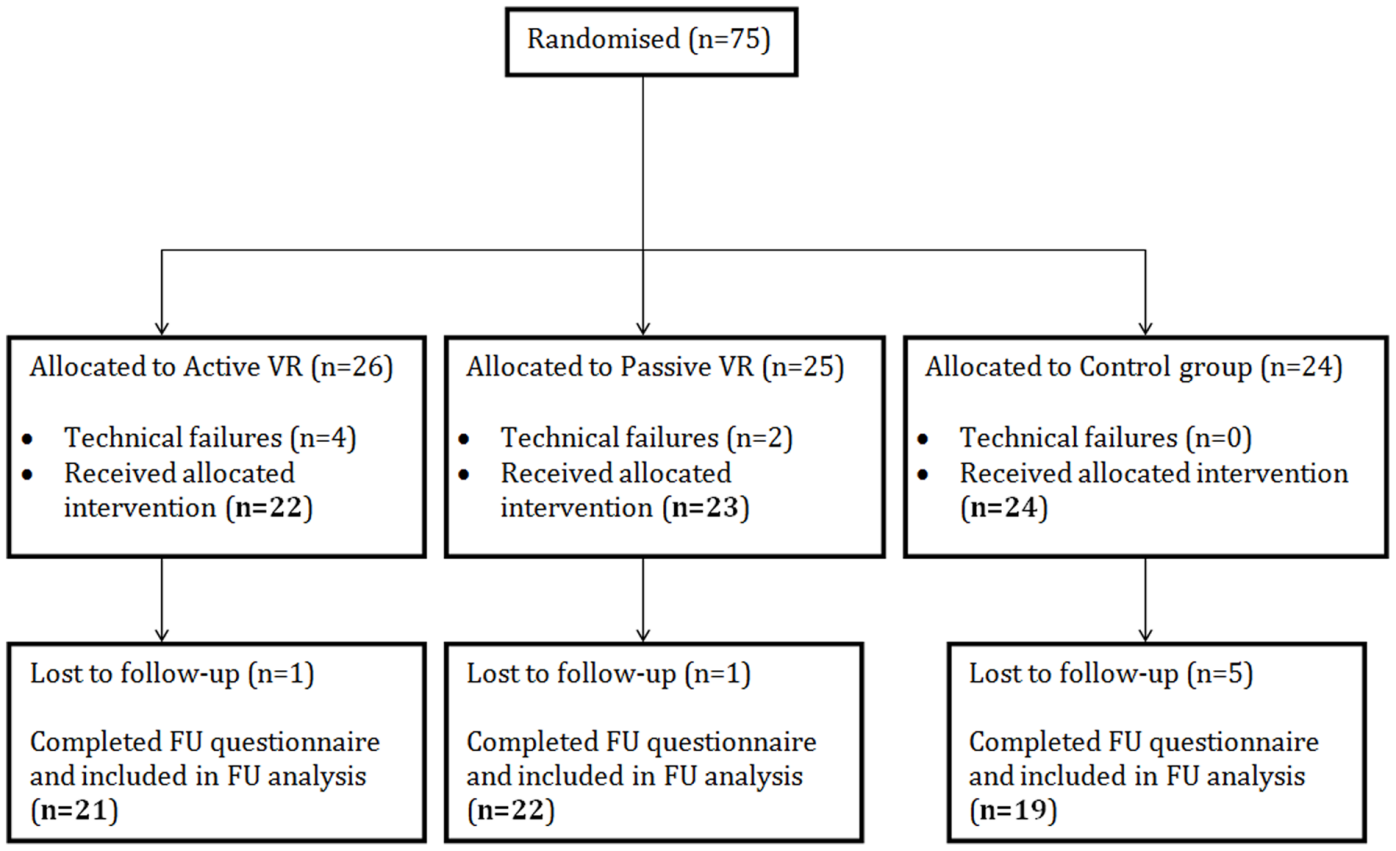
Flowchart of participants.

Seventy-five participants were recruited through a participant pool containing general public as well as university staff and students. They received £4 for their participation. Data from six participants were excluded because of technical failures (crashed VR environment; remote control stopped working) that required intervention from the experimenter, leaving data from 69 people (28 male, mean age = 33.1 years, SD = 12.7). A one-week follow-up telephone interview (mean = 7.13 days, SD = .42) collected data from 62 participants. We called participants up to 3 times within the set-up appointment time frame and sent an email to reschedule if they did not respond to the phone calls. Seven participants did not pick up their phone on any of the occasions or responded to the email so their follow-up data is missing. Of the seven participants who did not complete the follow-up part of the study, five (71%) were part of the control condition. Please refer to Figure 1 for the participant flow-chart.

Data on oral health characteristics showed that 29% of the participants had no fillings, 52% between 1 and 5 fillings, 16% between 6 and 10 fillings and 3% had more than ten fillings. One third of the study population had had at least one wisdom tooth removed. The last visit to the dentist was in the last month for 13% of the participants. Another 20% went 2–3 months ago, 13% 4–6 months ago, 28% 6–12 months ago, 16% 1–2 years ago and another 10% longer than 2 years ago.

### Design

Participants were exposed to one of three conditions in a fully randomised between participant design: Control no VR; Active VR; Passive VR). In addition participants were split into high vs. low Dental Anxiety based on their Dental Anxiety scores collected at the start of the study. This effectively produced a 3 (Condition: Control; Active VR; Passive VR) by 2 (Baseline Dental anxiety: High/Low) between participant design.

The difference between the active and the passive VR groups was that the first group was able to actively navigate the VR environment by using a controller. The passive group was a yoked control group; participants in this group watched a recording of the VR walk that the previous participant in the active condition generated. A total number of 22 walks were generated by the active participants and each of these walks was shown to a participant in the passive group. Taken together, both VR groups were thus shown the exact same content. Participants in the control group wore the head-mounted device (HMD) but only saw a black screen. In most research on VR distraction, a VR group is compared to a standard care situation (either between or within-subjects) [4], [8]. Although such a set-up allows for conclusions to be drawn regarding the effectiveness of VR distraction, it does not provide an answer to the question if it is the presence of the VR environment or the exclusion of the medical environment that accounts for the effect. In the current study we chose to include a black-screen control group to add this perspective and to be able to attribute the effects to the presence of a VR environment.

### Procedure

Participants completed an online dental anxiety questionnaire when they enrolled in the study, at least 24 hours prior to the experimental session.

#### Setting

**Figure 2:**
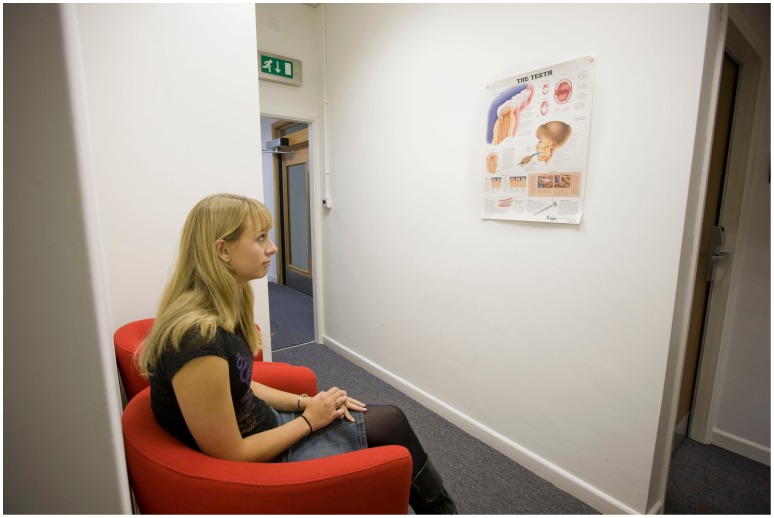
Set-up of the study. The person depicted in the images has given written informed consent, as outlined in the PLOS consent form, to publication of their photograph.

**Figure 3:**
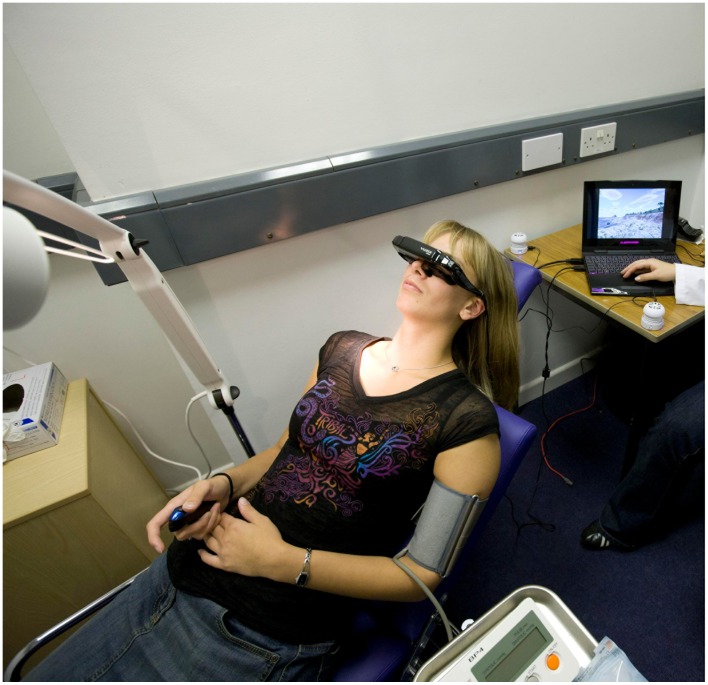
Set-up of the study. The person depicted in the images has given written informed consent, as outlined in the PLOS consent form, to publication of their photograph.

A simulated dental waiting and treatment area was created, using cues usually present in those areas. One part of the lab represented a waiting area with a row of chairs, and posters on the wall depicting dental information (see Figure 2). Here we took informed consent, collected baseline data and explained the procedure. A simulated treatment area was created in the other part of the lab (see Figure 3), with a dental chair, overhead light, dental instruments and a dentistry-related smell (drops of oil of cloves on cotton wool). The experimenter was wearing a white lab coat.

#### The simulated dental experience

Participants sat in the dental chair and listened to an audio tape of a dental treatment (performed by a practicing dentist), involving the administration of local anaesthetics, cavity preparation and filling, and an uncomplicated removal of a small upper wisdom tooth. They were asked to open their mouth during this simulated dental treatment and follow the instructions on the recording, for example ‘to open their mouth really wide’. They were reassured that their mouth would not be touched at any point. At baseline we measured heart rate and blood pressure. During the simulated treatment we measured heart rate, and immediately following treatment we measured blood pressure. Preliminary analysis found no significant differences in the temporal patterns of heart rate and blood pressure as a function of condition so physiological results are not considered further. Afterwards we collected measures on their experience of the event, the VR experience, demographic (age, gender and education) and background information (number of fillings, removal of a wisdom tooth, last and next dental visit, familiarity with the VR environment) with computer-based questionnaires. An appointment was made for a telephone call one week later and participants received their honorarium. Following research using the Elaborated Intrusions paradigm [26], [27], one week later intrusive thoughts and vividness of memories were measured and participants were debriefed.

#### Virtual environment and VR equipment

**Figure 4:**
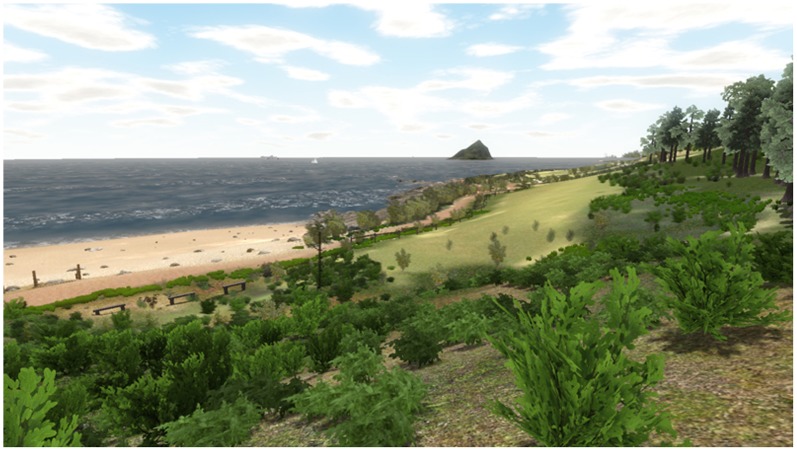
Screenshot of the VR environment.

The virtual environment (VE) depicted an existing environment, which consists of a coastal path, complete with sea, beach and field areas (see Figure 4), originally developed for restorative and rehabilitative environment studies [28]. The VE was constructed using commercially-sourced topographical geometry and aerial photographic images, and the resulting 3D model was used as a template to enable the VE to be populated with additional 3D assets and photographic textures, including the accurate representations of the few buildings at the site, trees, plants and other features.

A Vuzix iWear VR920 headset was connected to an Alienware M11X laptop (dual-core, 1.3GHz Intel processor with Nvidia GT 540M graphics card) and used to display the VE. The headset consists of two LCD displays with a 640×480 resolution, provides a 32-degree field of view and weighs 3.2 ounces. Head tracking of the HMD was switched off due to the context, since it would be inadvisable for the participant to move their head during dental treatment. Participants in the active condition were able to explore the VE in a first-person perspective, by using a Zeemote JS1 Thumbstick Controller. This controller was also used to look around.

### Measures

#### Moderator

*Dental Anxiety* was measured using the modified dental anxiety scale, which is often used in clinical practice to assess patients’ level of dental anxiety [29]. This 5-point scale, ranging from not anxious [1] to extremely anxious [5] contains 5 items and a sum score was calculated as in indicator of dental anxiety. Participants were divided into high-and low-dental anxiety groups based on a median split (median = 13, range 6–22), resulting in a low dental anxiety group of 37 participants scoring 6 to 13 (*M* = 9.76, *SD* = 2.23) and a high dental anxiety group of 32 participants scoring 14 to 22 (*M* = 17.06, *SD* = 2.26).

#### Immediate dental experience

*Compliance* with the four requests made by the dentist on the tape was recorded; participants were for example instructed to open their mouth really wide. This resulted in a score between two and four since there was no non-compliance amongst the participants. The sum score of the four items was used as a measure of compliance and totally compliant participants (scoring 4) were compared with not totally compliant participants (scoring 2 or 3).

To measure t*ime perception* participants were asked to estimate how long they thought the simulated treatment lasted for (actual time: 5 minutes and 43 seconds). The ratio of subjective duration to objective duration was calculated. A perfect estimation is indicated by a ratio of 1.0, whereas ratios higher than 1.0 indicate overestimation and ratios lower than 1.0 indicate underestimation.

#### VR experience

*Perceived control* (α = .66) was included as a manipulation check for the active versus passive VR manipulation using a scale based on the dominance dimension of the PAD-model [30]. This bipolar scale ranged from [1] to [9]. Sample items include “in control/controlled” and “guided/autonomous”.

Level of *presence* (α = .86) was assessed in both VR groups using six items selected from the IGroup Presence Questionnaire [31] and the Reality Presence Questionnaire [32] and the average score was calculated as in indicator of level of presence. An 11-point verbal rating scale, ranging from [1] to [11] was used and sample items include “I was completely captivated by the virtual world” and “How real did the virtual world seem to you?”.

Participants were also asked to indicate their *awareness of the surrounding environment* when wearing the HMD and to indicate to what extent they would choose to wear goggles or use VR during a real dental visit as a measure of behavioural intention. Both items were measured on an 11-point verbal rating scale, ranging from [1] to [11].

#### Follow-up dental experience

For the purpose of the current study we developed a questionnaire that assessed intrusive thoughts of the experience and vividness of memories of the experience. This questionnaire is based on the Alcohol Craving Experience Questionnaire [33] which was developed to measure vividness of memories and intrusive thoughts in a different context. We assessed whether participants suffered from *intrusive thoughts* about the experience (α = .81) and the *vividness of memories* (α = .69). Intrusive thoughts were assessed with two items on an 11-point verbal rating scale ranging from *not at all* [0] to *constantly/extremely* [10] and an average score was calculated. The items were “How often have you thought about the visit in the past week?” and To what extent did your thoughts about the visit pop into your mind spontaneously?”. The vividness of memories was measured with 5 items on an 11-point verbal rating scale ranging from *not at all* [0] to *extremely vividly* [10] and the average score was calculated. Sample items include “How vividly do you do you feel the emotions you experienced?”, “How vividly do you remember the discomfort of holding your mouth open?”, and “How vividly do you imagine the sounds?”.

### Statistical Procedure

A series of Analyses of Variance (ANOVA) with a 3 (condition: VR active, VR passive, control)×2 (dental anxiety: high, low) between-participant design with planned contrasts were carried out. The first contrast tested the difference between VR (both active and passive together) and the no VR control group. The second contrast tested the difference between the active and passive VR groups. Additionally, the interaction effects between VR condition and dental anxiety were examined to understand the role of dental anxiety. Degrees of freedom may vary across analyses due to the loss of participants at follow-up and not all measures being relevant for all groups in the study. A chi-square test was used for the not normally distributed data of the compliance measure.

## Results

Table 1 includes the means and standard deviations for the three groups on the different outcome measures. All met assumptions of normality with acceptable skewness and kurtosis apart from compliance, which was high with 75% of all participants complying with all four requests, and no-one missing more than two requests.

**Table 1:** Overview of the means and standard deviations (between brackets) for the dependent variables.

DV	Active VR	Passive VR	No VR control
	(n = 22)	(n = 23)	(n = 24)
Perceived control	3.94 (1.57)	3.13 (1.20)	N/A
Compliance	3.67 (.66)	3.95 (.21)	3.57 (.66)
- Low dental anxiety	3.60 (.70)	4.00 (.00)	3.71 (.47)
- High dental anxiety	3.73 (.65)	3.90 (.32)	3.33 (.87)
Time perception (ratio)	1.33 (.50)	1.24 (.48)	1.31 (.57)
- Low dental anxiety	1.12 (.40)	1.22 (.55)	1.26 (.54)
- High dental anxiety	1.51 (.51)	1.35 (.46)	1.39 (.63)
Presence	6.21 (1.51)	5.16 (1.65)	N/A
- Low dental anxiety	5.43 (1.04)	5.27 (1.44)	
- High dental anxiety	6.86 (1.57)	4.92 (1.89)	
Awareness of the surrounding environment	4.05 (2.36)	4.61 (2.21)	5.17 (2.48)
- Low dental anxiety	4.10 (2.23)	4.85 (2.15)	4.73 (2.71)
- High dental anxiety	4.00 (2.56)	4.45 (2.30)	5.89 (1.97)
Interest in using VR during real dental visit	8.59 (1.94)	8.09 (2.41)	6.92 (2.78)
- Low dental anxiety	7.90 (2.28)	7.31 (2.96)	6.47 (2.80)
- High dental anxiety	9.17 (1.47)	8.82 (1.17)	7.67 (2.74)
*Sample sizes for follow-up measures*	*n = 21*	*n = 22*	*n = 19*
Intrusive thoughts	1.68 (.98)	1.83 (1.26)	1.61 (1.25)
- Low dental anxiety	1.37 (.70)	1.31 (1.36)	1.49 (1.41)
- High dental anxiety	1.92 (1.12)	2.40 (.80)	1.89 (.86)
Vividness of memories	4.26 (.88)	4.40 (1.40)	4.55 (2.23)
- Low dental anxiety	4.13 (1.01)	4.28 (1.29)	3.77 (2.12)
- High dental anxiety	4.35 (.79)	4.34 (1.69)	6.23 (1.46)

### Baseline Characteristics

No baseline differences between the experimental groups were found regarding participants’ demographic variables, oral health characteristics, and familiarity with the VR environment, all *p*s >.05.

### Immediate Dental Experience

Comparing totally compliant and not totally compliant participants, the passive group were most compliant with only one person not being totally compliant; five participants in the active group and eight in the control group missed one or two requests (χ^2^(2) = 6.27, *p* = .043). No moderating effect of dental anxiety was present, all *p*s >.05. No effects of VR condition were found on time perception (*F*<1), but the main effect of dental anxiety approached significance (*F*(1,63) = 3.76, *p* = .057, *η*_p_^2^ = .06). Participants with higher dental anxiety made a larger overestimation (*M* = 1.42, *SD* = .52) than those with lower dental anxiety (*M* = 1.18, *SD* = .48). While the actual time of the treatment was 5.7 minutes, participants with high dental anxiety estimated it lasted for 8.1 (*SD = *3.0) minutes and participants with lower dental anxiety estimated 6.8 (*SD = *2.7) minutes.

### VR Experience

The manipulation check of perceived control showed that participants in the active VR group experienced a higher level of control than those in the passive VR group (*F*(1,66) = 4.38, *p* = .040, *η*_p_^2^ = .06).

**Figure 5:**
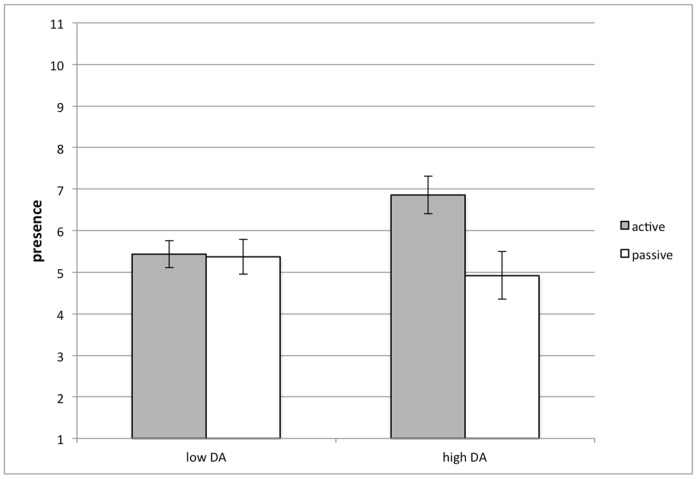
The interaction effect of VR and dental anxiety on feelings of presence.

The active VR group experienced a higher level of presence than the passive VR group (*F* (1,41) = 4.77, *p = *.035, *η*_p_^2^ = .10). An interaction between VR condition and dental anxiety was found (*F* (1,41) = 4.23, *p = *.046, *η*_p_^2^ = .09). Participants with a higher level of dental anxiety felt more presence in the VR if they could actively control it (*M* = 6.86, *SD* = 1.57) than if they were passively watching it (*M* = 4.92, *SD* = 1.89; *F*(1,41) = 9.22, *p* = .004, *η*_p_^2^ = .18; see Figure 5).

Although the results for participants’ awareness of the surrounding environment were in the expected direction, with the active VR group being the least aware, the passive VR group slightly more aware and the control group most aware, these differences did not reach statistical significance and we found no interaction effect for dental anxiety (*F*<1).

Participants were asked to indicate to what extent they would choose to wear goggles or use VR during a real dental visit. Participants in the VR groups were more interested to use VR during a dental visit than participant in the control group (*F* (1,63) = 4.19, *p = *.045, *η*_p_^2^ = .06). And more importantly, we also found a main effect of dental anxiety. Participants with more dental anxiety (*M* = 8.63, *SD* = 1.88) were more interested to use VR during real dental treatment than those with lower levels of dental anxiety (*M* = 7.16, *SD* = 2.75; *F* (1,63) = 4.93, *p = *.030, *η*_p_^2^ = .07).

### Follow-up Dental Experience

No effects were found on the intrusive thoughts participants experienced as a consequence of VR distraction (F<1), or on vividness of memories (*F*(1,56) = 2.55, *p* = .12). A main effect was found for dental anxiety (*F* (1,56) = 4.89, *p = *.031, *η*_p_^2^ = .08) on intrusive thoughts. Participants with more dental anxiety (*M* = 3.10, *SD* = 1.44) experienced more intrusive thoughts than those with lower levels of dental anxiety (*M* = 2.11, *SD* = 1.84). No interaction effect for dental anxiety was found (*F*<1). A main effect for dental anxiety was also found (*F* (1,56) = 4.92, *p = *.031, *η*_p_^2^ = .08) for vividness of memories.

**Figure 6:**
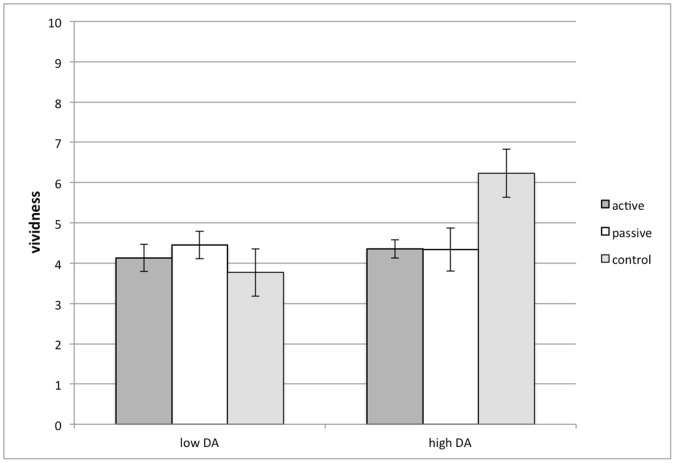
The interaction effect of VR and dental anxiety on vividness of memories.

Most importantly, a significant interaction between VR condition and dental anxiety was found for vividness of memories (*F* (2,56) = 4.06, *p = *.023, *η*_p_^2^ = .13). Simple main effect analysis showed that for participants with higher dental anxiety, both active (*M* = 4.35, *SD* = .79) and passive VR (*M* = 4.34, *SD* = 1.69) distraction resulted in less vivid memories compared to the black-screen control group (*M* = 6.23, *SD* = 1.46; *F*(2,56) = 3.89, *p* = .026, *η*_p_^2^ = .12; see Figure 6). This shows that VR was successful at interrupting the memory process in particular for highly anxious participants.

## Discussion

Our research extends previous VR studies by showing that VR distraction in a simulated dental context affected memories a week later. The VR distraction had effects not only on concurrent experiences, but also longitudinally upon the vividness of memories after the dental experience had ended. Participants higher in dental anxiety (for whom the procedures were presumably more aversive) showed a greater reduction in memory vividness than lower dental-anxiety participants. This is an important extension because it helps us understand the cognitive processes by which VR distraction can work.

Dental anxiety is associated with the tendency to experience negative or threatening thoughts concerning treatment [34] and this may prevent patients arranging and attending dental appointments. Our findings suggest that VR distraction has the potential to influence people’s memories of a potentially anxiety-inducing medical event. Our results are promising for real dental procedures in suggesting that VR distraction during dental treatment has the potential to interrupt the cycle of dental anxiety [14], by blocking the development of vivid memories.

It is important to note that the current study took place in a simulated environment. We chose a simulated rather than real treatment for ethical reasons, as we wanted to include participants high in dental anxiety, for whom a simulated treatment would be stressful already. And whilst we do find differential effects for participants high and low in dental anxiety, there were no differences on the physiological measures between these two groups. This does mean that we are currently unable to draw any conclusions regarding the effectiveness of VR distraction during real dental treatment. As with any experimental study, there always is the worry about demand characteristics. We do however find a moderating effect on dental anxiety, and do not think it likely that people with higher or lower dental anxiety would differ in their desire to comply with an experimenter. Next, we collected the measure of vividness of memories one week later. If demand characteristics really were at play in this study, we would expect a lot of participants to still remember all the details and what we would possibly want them to answer. Also, we would presume that demand characteristics would play a greater role in within-subject designs where participants are exposed to all conditions, while the current study employed a between-subjects design.

Frere and colleagues [20] suggest that the use of VR equipment will be particularly useful for long procedures or treatment of patients who have to have repeated procedures. In order to realize cost-effective VR distraction interventions, it would be desirable to identify those patients that will most benefit from this. Our findings suggest that anxious patients, rather than being resistant to distraction interventions, would be most likely to benefit from VR. Interestingly, participants with more dental anxiety were also more interested to use VR during real dental treatment than those with lower levels of dental anxiety, and especially participants with a higher level of dental anxiety felt more presence in the VR if they could actively control it than if they were passively watching a recording. These results are in line with the ideas about how anxiety influences attention [35] and suggest that VR distraction, or possibly any distraction intervention, could be particularly suitable for this high anxiety group. We recognise that no real-time recordings of anxiety were gathered during the simulated treatment, primarily to avoid the participant having to disengage with the immersive scenario, and thus we are unable to comment on the temporal patterns in anxiety during VR distraction. Future research could monitor how anxiety might be affected at different stages during treatment.

Previous research found that interactive VR was better than passive VR in children experiencing experimentally induced pain [5], [36]. Our participants in the active group experienced a higher level of control and presence, and participants in the passive VR group were more compliant than active and control participants, yet active versus passive VR had no effects on immediate outcomes or a week later. More research is needed to decide whether this is because we used a calming natural environment that people simply walked around in (rather than an interactive game), or whether this was due to our simulated context or adult sample. Most research in the domain of VR distraction made use of existing video games as the distractor, e.g. [5], [37], or games developed for the purpose of using it as a VR distractor [38], [39]. Both types of games have proven to be effective distractors, but it is unclear whether gaming elements, such as providing a goal, are required for a VR distraction intervention to be effective.

A variety of other imagery and stimuli has been used to distract patients in previous research including natural contexts such as forests [40] and a botanical garden [21]. Research on restorative environments suggests that certain environments are capable of relaxing people, especially natural environments [41]. Hence we would call for more research that addresses the content of VR interventions to help us understand which specific elements are successful.

The cognitive effects were measured at one-week follow-up, following the Elaborated Intrusions paradigm. One might argue that a one-week follow up assessment of memories does not reflect the amount of time that is usually present between dental appointments. While it is not the most common situation, a variety of treatments do require patients to return a week later for the next part of their treatment, for example when crowns or dentures are needed. Also, the current study only offered a first test of this elaborated intrusions account, so it did seem prudent to test the effect at one week follow-up first before investing in studies with a more longitudinal character. Arguably, the week immediately following such an experience is crucial for consolidating and processing any relevant memories.

One of the claims that is often made for the usage of VR as a distraction technique is that wearing a HMD effectively excludes the surrounding medical environment. For example, the appearance of the nurse who cleans patient’s wounds may be a strong enough cue to create anxiety [38]. The overhead light and the dental instruments may induce anxiety in a similar way even in a simulated context. In the current study we chose to include a black-screen control group to add this perspective and to be able to attribute the effects to the presence of a VR environment. However, further research is needed to decide if it is the presence of the VR environment or the exclusion of the medical environment that accounts for the effect.

Taken together, the current study provides evidence that a VR distraction intervention can not only impact the experience of a simulated aversive event, it can also reduce the vividness of memories of such an event a week later. This study thus suggests that VR distractions can be considered as a relevant intervention for cycles of care in which people’s previous experiences affect their behaviour for future events. If a dental patient for example has a more positive experience of a treatment due to the VR distraction intervention, that patient might have less vivid memories and as a consequence might be less likely to postpone a future dental visit.

## References

[1] Maggirias J, Locker D (2002) Psychological factors and perceptions of pain associated with dental treatment. Community Dent Oral Epidemiol 30: 151–159. 12000356 10.1034/j.1600-0528.2002.300209.x

[2] Bare LC, Dundes L (2004) Strategies for combating dental anxiety. J Dent Educ 68: 1172–1177. 15520236

[3] McCaul KD, Monson N, Maki RH (1992) Does distraction reduce pain-produced distress among college students? Health Psychol 11: 210–217. 1396488 10.1037//0278-6133.11.4.210

[4] Mahrer N, Gold J (2009) The use of virtual reality for pain control: A review. Cur Pain Headache Rep 13: 100–109. 10.1007/s11916-009-0019-8 19272275

[5] Dahlquist LM, McKenna KD, Jones KK, Dillinger L, Weiss KE, et al (2007) Active and passive distraction using a head-mounted display helmet: effects on cold pressor pain in children. Health Psychol 26: 794–801. 18020853 10.1037/0278-6133.26.6.794

[6] Leibovici V, Magora F, Cohen S, Ingber A (2009) Effects of virtual reality immersion and audiovisual distraction techniques for patients with pruritus. Pain Res Manag 14: 283–286. 19714267 10.1155/2009/178751 PMC2734514

[7] Schneider SM, Hood LE (2007) Virtual reality: A distraction intervention for chemotherapy. Oncol Nurs Forum 34: 39–46. 17562631 10.1188/07.ONF.39-46 PMC2121303

[8] Malloy KM, Milling LS (2010) The effectiveness of virtual reality distraction for pain reduction: A systematic review. Clin Psychol Rev 30: 1011–1018. 20691523 10.1016/j.cpr.2010.07.001

[9] Gold JI, Belmont KA, Thomas DA (2007) The neurobiology of virtual reality pain attenuation. CyberPsychol Behav 10: 536–544. 17711362 10.1089/cpb.2007.9993

[10] Oosterink FMD, de Jongh A, Hoogstraten J (2009) Prevalence of dental fear and phobia relative to other fear and phobia subtypes. Eur J Oral Sci 117: 135–143. 19320722 10.1111/j.1600-0722.2008.00602.x

[11] Kleinknecht RA, Bernstein DA (1978) The assessment of dental fear. Behav Ther 9: 626–634.

[12] Locker D, Liddell AM (1991) Correlates of dental anxiety among older adults. J Dent Res 70: 198–203. 1999559 10.1177/00220345910700030801

[13] Hill KB, Hainsworth JM, Burke FJT, Fairbrother KJ (2008) Evaluation of dentists’ perceived needs regarding treatment of the anxious patient. Br Dent J 204: E13. 18425075 10.1038/sj.bdj.2008.318

[14] Armfield J, Stewart J, Spencer AJ (2007) The vicious cycle of dental fear: exploring the interplay between oral health, service utilization and dental fear. BMC Oral Health 7: 1. 17222356 10.1186/1472-6831-7-1 PMC1784087

[15] Redelmeier DA, Katz J, Kahneman D (2003) Memories of colonoscopy: a randomized trial. Pain 104: 187–194. 12855328 10.1016/s0304-3959(03)00003-4

[16] Kavanagh DJ, Andrade J, May J (1995) Imaginary relish and exquisite torture: The Elaborated Intrusion theory of desire. Psychol Rev 112: 446–467. 10.1037/0033-295X.112.2.446 15783293

[17] Brewin CR, Dalgleish T, Joseph S (1996) A dual representation theory of posttraumatic stress disorder. Psychol Rev 103: 670–686. 8888651 10.1037/0033-295x.103.4.670

[18] Wegner DM, Scheider DJ, Carter SR, White TL (1987) Paradoxical effects of thought suppression. J Pers Soc Psychol 53: 5–13. 3612492 10.1037//0022-3514.53.1.5

[19] Hoffman HG, Garcia-Palacios A, Patterson DR, Jensen M, Furness T, et al (2001) The effectiveness of virtual reality for dental pain control: A case study. CyberPsychol Behav 4: 527–535. 11708732 10.1089/109493101750527088

[20] Frere CL, Crout R, Yorty J, McNeil DW (2001) Effects of audiovisual distraction during dental prophylaxis. J Am Dent Assoc 132: 1031–1038. 11480629 10.14219/jada.archive.2001.0309

[21] Furman E, Jasinevicius TR, Bissada NF, Victoroff KZ, Skillicorn R, et al (2009) Virtual reality distraction for pain control during periodontal scaling and root planing procedures. J Am Dent Assoc 140: 1508–1516. 19955069 10.14219/jada.archive.2009.0102

[22] Beukeboom CJ, Langeveld D, Tanja-Dijkstra K (2012) Stress-reducing effects of real and artificial nature in a hospital waiting room. J Altern Complement Med 18: 329–333. 22489806 10.1089/acm.2011.0488

[23] Dijkstra K, Pieterse ME, Pruyn A (2008) Stress-reducing effects of indoor plants in the built healthcare environment: The mediating role of perceived attractiveness. Prev Med 47: 279–283. 18329704 10.1016/j.ypmed.2008.01.013

[24] Wheeler B, White MP, Stahl-Timmins W, Depledge MH (2012) Does living by the coast improve health and wellbeing? Health Place 18: 1198–1201. 22796370 10.1016/j.healthplace.2012.06.015

[25] Volker S, Kistemann T (2011) The impact of blue space on human health and well-being – Salutogenic health effects of inland surface waters: A review. Int J Hyg Environ Health 214: 449–460. 21665536 10.1016/j.ijheh.2011.05.001

[26] Krans J, Näring G, Holmes EA, Becker ES (2010) “I see what you’re saying”: Intrusive images from listening to a traumatic verbal report. J Anxiety Disord 24: 134–140. 19864108 10.1016/j.janxdis.2009.09.009

[27] Holmes EA, Brewin CR, Hennessy RG (2004) Trauma films, information processing, and intrusive memory development. J Exp Psychol Gen 133: 3–22. 14979748 10.1037/0096-3445.133.1.3

[28] Knight JF, Stone RJ, Qian C (2012) Virtual restorative environments: preliminary studies in scene, sound and smell. International Journal of Gaming and Computer-Mediated Simulations 2012 4: 3.

[29] Humphris GM, Morrison T, Linsay SJE (1995) The Modified Dental Anxiety Scale: validation and United Kingdom norms. Community Dent Health12: 143–150. 7584581

[30] Mehrabian A, Russell JA (1974) An approach to environmental psychology. M.I.T. Press, Cambridge, Mass.

[31] Schubert T, Friedmann F, Regenbrecht H (2001) The experience of presence: Factor analytic insights. Presence 10: 266–281.

[32] Baños RM, Botella C, Garcia-Palacios A, Villa H, Perpina C, et al (2000) Presence and reality judgment in virtual environments: A unitary construct? CyberPsychol Behav 3: 327–335.

[33] Statham DJ, Connor JP, Kavanagh DJ, Feeny GFX, Young RMD, et al (2011) Measuring alcohol craving: development of the Alcohol Craving Experinece Questionnaire. Addict106: 1230–1238. 10.1111/j.1360-0443.2011.03442.x 21438940

[34] De Jongh A, ter Horst G (1995) Dutch students’ dental anxiety and occurance of thoughts related to treatment. Community Dent Oral Epidemiol 23: 170–172. 7634773 10.1111/j.1600-0528.1995.tb00223.x

[35] Derakshan N, Eysenck MW (2009) Anxiety, processing efficiency, and cognitive performance. New developments from attentional control theory. Eur Psychol 14: 168–176.

[36] Law EF, Dahlquist LM, Sil S, Weiss KE, Herbert LJ, et al (2011) Videogame distraction using virtual reality technology for children experiencing cold pressor pain: The role of cognitive processing. J Pediatr Psychol 36: 84–94. 20656761 10.1093/jpepsy/jsq063 PMC3107585

[37] Schneider SM, Ellis M, Coombs WT, Shonkwiler EL, Folsom LC (2003) Virtual reality intervention for older women with breast cancer. CyberPsychol Behav 6: 103–107. 10.1089/109493103322011605 PMC3645300 12855087

[38] Hoffman HG, Doctor JN, Patterson DR, Carrougher GJ, Furness TA III (2000) Virtual reality as an adjunctive pain control during burn wound care in adolescent patients. Pain 85: 305–309. 10692634 10.1016/s0304-3959(99)00275-4

[39] Schmitt YS, Hoffman HG, Blough DK, Patterson DR, Jensen MP, et al (2011) A randomized, controlled trial of immersive virtual reality analgesia, during physical therapy for pediatric burns. Burns 37: 61–68. 20692769 10.1016/j.burns.2010.07.007 PMC2980790

[40] Mühlberger A, Wieser MJ, Kenntner-Mabiala R, Pauli P, Wiederhold BK (2007) Pain modulation during drives through cold and hot virtual environments. CyberPsychol Behav 10: 516–522. 17711359 10.1089/cpb.2007.9996

[41] Hartig T, Böök A, Garvill J, Olsson T, Gärling T (1996) Environmental influences on psychological restoration. Scand Journal Psychol 37: 378–393. 10.1111/j.1467-9450.1996.tb00670.x 8931393

